# Anti-hypothalamus autoantibodies in anorexia nervosa: a possible new mechanism in neuro-physiological derangement?

**DOI:** 10.1007/s40519-022-01388-5

**Published:** 2022-03-16

**Authors:** Andrea Escelsior, Ludovica Cogorno, Samir G. Sukkar, Andrea Amerio, Lorenzo M. Donini, Marina Bellomo, Erika Iervasi, Mario Amore, Daniele Saverino

**Affiliations:** 1 Department of Neuroscience, Rehabilitation, Ophthalmology, Genetics, Maternal and Child Health (DINOGMI), Section of Psychiatry, University of Genoa, 16132 Genoa, Italy; 2 IRCCS Ospedale Policlinico San Martino, 16132 Genoa, Italy; 3 Dietetics and Clinical Nutrition Unit, Genoa University, 16132 Genoa, Italy; 4 Experimental Medicine Department, Medical Pathophysiology, Food Science and Endocrinology Section, Food Science and Human Nutrition Research Unit, Sapienza University, Rome, Italy; 5 Department of Experimental Medicine (DiMeS), Section of Human Anatomy, University of Genoa, Via De Toni, 14, 16132 Genoa, Italy

**Keywords:** Anorexia nervosa, Hypothalamic autoantibodies, Orexigenic molecules, Anorexigenic molecules, Autoimmunity

## Abstract

**Purpose:**

Anorexia nervosa (AN) is a serious and complex mental disorder affecting mainly young adult women. AN patients are characterized by low body weight in combination with self-induced starvation, intense fear of gaining weight, and distortion of body image. AN is a multifactorial disease, linked by recent evidence to a dysregulation of the immune system.

**Methods:**

In this pilot study, 22 blood serums from AN patients were tested for the presence of autoantibodies against primate hypothalamic periventricular neurons by immunofluorescence and by a home-made ELISA assay. Cellular fluorescence suggests the presence of autoantibodies which are able to recognize these neurons (both to body cell and fiber levels). By means of ELISA, these autoantibodies are quantitatively evaluated. In addition, orexigenic and anorexigenic molecules were measured by ELISA. As control, 18 blood serums from healthy age matched woman were analysed.

**Results:**

All AN patients showed a reactivity against hypothalamic neurons both by immunofluorescence and ELISA. In addition, ghrelin, pro-opiomelanocortin (POMC), and agouti-related peptide (AGRP) were significantly higher than in control serums (*p* < 0.0001). In contrast, leptin was significantly lower in AN patients than controls (*p* < 0.0001).

**Conclusions:**

Immunoreaction and ELISA assays on AN blood serum suggest the presence of autoantibodies AN related. However, it is not easy to determine the action of these antibodies in vivo: they could interact with specific ligands expressed by hypothalamic cells preventing their physiological role, however, it is also possible that they could induce an aspecific stimulation in the target cells leading to an increased secretion of anorexigenic molecules. Further studies are needed to fully understand the involvement of the immune system in AN pathogenesis.

**Level of evidence:**

V, descriptive study.

## Introduction

Anorexia nervosa (AN) is a serious and complex mental disorder affecting mainly young adult women [1, 2]. AN patients are characterized by significant low body weight in combination with self-induced starvation, intense fear of gaining weight and distortion of body image [3].

One of the key points characterizing AN patients is to chronically reduce food/energy intake, with severe malnutrition often protracted for several years, strictly correlated to morbidity and mortality due to the failure in a lot of organs [4].

It seems that an alteration in the communication between the periphery and the brain exists: there might be an inability of the brain to translate the circulating factors that signal and regulate the energy state of the organism in the proper and coherent adaptive behaviour [5].

It is possible that AN is a multifactorial disease. Recent genomic association studies provide evidence of a genetic predisposition for a body weight set point in AN [6–9]. In this framework, AN could be not only a psychiatric disorder but also a metabolic one [10].

Several studies focused on the morpho-functional characteristics of the brain using functional magnetic resonance imaging [11]. Otherwise, the neuro-physiological control of food intake and energy control in eating disorders and especially in AN is not completely understood [11].

The hypothalamus has got a crucial role in feeding behaviour and contains the major centres that regulate eating [12]. The arcuate nucleus is involved in the long-term regulation of appetite and body weight [12]. Here, leptin, insulin, ghrelin, and other peripheral hormones act on arcuate neurons that seem to play antagonistic roles in energy balance control [12].

It has recently been speculated that AN may be a disease linked to a dysregulation of the immune system [13–16]. A possible role of molecular mimicking has been hypothesized: early exposure to pathogens induces the production of autoantibodies specific for neuropeptides, neurotransmitters and peptide hormones regulating appetite [13]. Consequently, hypothalamic neurons modify appetite and mood, leading to a decrease in food intake [13–16].

In addition, a higher prevalence of autoimmune diseases such as type 1 diabetes and Crohn’s disease were observed among patients with eating disorders [14]. Otherwise, the reverse relationship is also suggested: patients with AN might be more susceptible to autoimmune diseases [15]. Finally, morbid fear, anxiety, and even amenorrhea could be a direct effect of the switching on or off of neuronal receptors in the hypothalamus [16]. Thus, the autoimmune hypothesis would not contradict existing theories involving psychological and cultural influences in AN but would instead improve our understanding of how they arise and act [16].

Searching for possible mechanisms implicating hypothalamic neurons in the aetiology and pathogenesis of AN, the aim of this study was to verify if hypothalamic systems, responsible for the regulation of food intake, could be targeted by autoantibodies in AN patients.

## Materials and methods

### Sample characteristics

AN patients were recruited from October 2019 to March 2021. Sixteen had restrictive anorexia, while 6 purging phenotype (vomiting or use of laxatives). The diagnosis was based on DSM‐V‐TR (Diagnostic and Statistical Manual of Mental Disorders, Fifth Edition, Text Revision) [17]. Blood samples were collected in the morning, between 7, 30 and 9, 30, before breakfast (and previous meal was at least 6 h earlier). All AN patients were women, aged from 14 to 62 years old (mean 24 ± 11.2 St. Dev.), with a BMI of 16.18 ± 2.04 (Table 1).

**Table 1:** Demographic and baseline characteristics of AN study groups

	Age	BMI	Ghrelin (pg/ml)	POMC (pg/ml)	ARGP (pg/ml)	Leptin (pg/ml)	IFI
1	17	17.00	452.73	768.75	960.50	2535.05	3
2	62	13.42	347.92	247.86	683.38	5216.34	3
3	18	18.90	280.16	71.55	1882.79	790.27	3
4	15	13.84	262.41	187.65	982.77	6378.84	3
5	24	17.95	451.87	251.06	261.09	4751.69	3
6	35	12.73	1030.26	330.84	348.22	3910.95	3
7	16	15.80	456.28	928.21	1372.75	741.08	3
8	14	18.50	592.50	685.33	154.97	2323.96	3
9	18	15.00	1199.99	742.57	713.20	4363.64	3
10	23	14.72	350.38	452.66	569.46	4055.31	2
11	25	16.73	506.13	178.45	471.43	2811.59	3
12	43	17.32	617.88	413.40	340.24	3246.50	3
13	18	14.30	373.66	322.32	434.42	4053.68	3
14	16	17.93	520.73	299.50	615.52	3458.33	2
15	33	12.98	476.21	335.92	399.25	2166.41	2
16	17	17.74	533.54	321.09	619.19	2870.32	2
17	28	17.12	795.21	407.86	395.88	3214.47	2
18	26	18.17	827.09	112.56	423.09	2151.27	3
19	25	18.70	740.64	109.16	884.63	2119.89	3
20	23	13.27	820.76	219.46	503.45	2009.47	3
21	18	17.37	472.91	301.34	528.47	2592.73	3
22	18	16.54	399.02	294.67	347.73	2610.74	3

The control group matched by gender and age with the group of AN patients consisted of 18 healthy women aged from 14 to 51 years old (mean 26 ± 9.2 St. Dev.) and with a BMI of 19.92 ± 0.43 (Table 2) not affected by any kind of eating disorders or by autoimmune diseases.

**Table 2:** Demographic characteristics and baseline characteristics of healthy control subjects

	Age	BMI	Ghrelin (pg/ml)	POMC (pg/ml)	ARGP (pg/ml)	Leptin (pg/ml)	IFI
1	30	25.00	223.98	102.14	29.68	14,032.41	0
2	25	19.13	228.18	165.42	4.75	13,458.74	0
3	22	22.33	193.28	69.32	20.40	13,978.92	0
4	24	20.65	115.40	61.56	16.68	20,281.91	0
5	28	24.90	201.32	51.92	3.46	14,015.27	0
6	30	22.45	276.43	231.17	54.98	8780.03	0
7	24	18.92	205.38	14.96	4.53	15,137.48	1
8	47	20.20	240.49	27.48	81.64	12,919.33	0
9	22	17.13	194.35	47.46	85.11	13,723.39	1
10	27	17.50	216.27	111.36	75.47	15,904.36	0
11	51	16.90	234.06	96.98	51.66	9578.84	0
12	14	16.79	217.40	78.614	115.36	16,953.43	0
13	24	16.71	204.32	66.63	97.28	17,563.74	0
14	15	19.68	200.43	85.73	178.17	19,171.65	0
15	24	18.21	250.02	97.53	72.06	15,790.48	0
16	25	22.04	198.45	78.92	81.23	15,729.83	0
17	26	20.74	219.29	69.10	78.21	14,722.81	0
18	24	19.30	293.02	92.18	77.92	15,283.11	0

Blood serums were stored frozen until the use. Freezing and thawing was avoided.

All patients provided written informed consent. Study was conducted in accordance with the Declaration of Helsinki II.

### Immunofluorescence

The detection of anti-hypothalamic autoantibodies was performed with the IIF method using kits provided free of charge by Euroimmun AG (Lubeck, Germany) [18]. In detail, each sample was analysed at a serum dilution of 1:10 on two different sections of primate (monkey) hypothalamic periventricular area [19]. Fluorescent anti-human immunoglobulins class G (IgG) conjugate was used. Positive and negative control serum was analysed in each analytical session. The intensity of the fluorescence was determined by two different readers blind to subject characteristics and an arbitrary scale from 0 to 3 was assigned (0 no reaction, 1 uncertain fluorescence, 2 nuclear fluorescence, 3 fibers and/or nuclear fluorescence). Samples scored positive if a 2 or 3 fluorescence reaction was observed.

### Anti-hypothalamus autoantibody ELISA protocols

In addition to immunofluorescence, the levels of autoantibodies (IgG and IgM) specific for hypothalamic antigens were measured in the serum of AN patients and healthy controls by a direct ELISA method built in our laboratory [20, 21]. Briefly, ninety-six-well Maxisorb flat-bottom plates (Nunc) were coated overnight at 4° C with an available bovine hypothalamic lysate (Science Cell Research, cat#0613) (10 µg/ml in 50 µl/well). After washings, plates were incubated for 2 h with phosphate buffered saline (PBS) -3% bovine serum albumin (BSA) to avoid aspecific interactions. After three washes, 100 µl of 1:100 in PBS-3% BSA diluted serum samples were plated in triplicate and incubated overnight at 4 °C with agitation. After washings, wells were incubated with 100 µl of anti-human IgG or IgM HRP-conjugate (Jackson Immune research, as 1:10,000 in PBS-3% BSA buffer) for 45 min at room temperature. Noteworthy, the secondary antibody used is specific to human IgG, to highlight the totality of autoantibodies to hypothalamic antigens. After incubation, plates were washed and 100 µl of tetramethylbenzidine (TMB) substrates were added to develop colour for 15 min. Stop Solution (100 µl) was then added, and plates were read at 450 nm for 15–20 min.

**Fig. 1:**
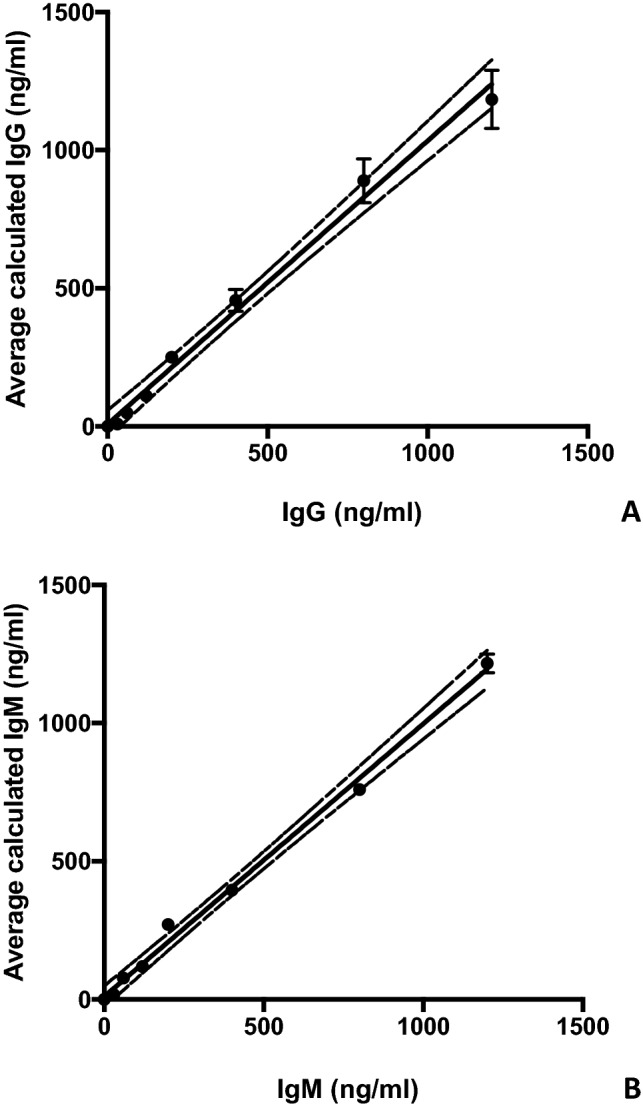
IgG and IgM autoantibody standard curves. IgG and IgM autoantibodies ELISA standard curves used to calculate the anti-hypothalamus antibodies concentrations in human subject serum. In (**A**) IgG standard curve and in (**B**) IgM standard curve are presented. Standard deviation (STD) from three independent runs were shown. Linear regression fitting results (continuous line) and 95% confidence bands of the best-fit line (dotted line) are shown

As a part of the assay, standard curves for human IgG and IgM were carried out (Fig. 1). It is important to note that these standard curves were not specific for hypothalamic antigens, but exclusively for IgG or IgM. Standard curves were obtained by adding 0, 30, 60, 120, 200, 400, 800, and 1200 ng/ml (50 µl) of purified human IgG (Sigma Co. cat# I4506) or human IgM (Sigma Co. cat#I8260) to the first three columns of the ELISA plates (see Fig. 1). Standards were used on the same plate when anti-hypothalamic reactivity levels for serum samples assaying were tested. Upon stopping and washing as described above, anti-human IgG HRP-conjugate (1:10,000 in PBS-3% BSA buffer) was added, followed by TMB substrate addition. Thus, optical density (absorbance) readings of serums from AN patients and healthy donors reflect the presence of human IgG against hypothalamic antigens. Figure 1 represents the standard curve for IgG by plotting the added IgG concentrations (x-axis) versus calculated IgG concentrations based on absorbance (y-axis) values (mean–standard deviation [SD] from three independent runs). Linear regression was performed with a regression coefficient R2 > 0.9917 (Fig. 1). This standard curve plot shows a reliable predictability of IgG concentrations (based on IgG amount recovery) as well as a good robustness (based on the low SD values over the whole range of the standard curves). These readings were converted into IgG concentration in µg/ml, applying a dilution factor of 50 to determine the anti-hypothalamic autoantibodies serum concentrations (50 µl of standards were used, while 1 µl of human serum per sample was loaded per assay). Intra-assay % coefficient of variation (CV) was 5–10% (for both IgG and IgM), and inter-assay % CV was 8.4–11.3% and 12.8–19.8% (respectively, for IgG and IgM). Finally, deviation between triplicates was < 10% for any reported value.

### ELISA

Specific ELISA kits were used for measuring different serum parameters, according to the manufacturer’s protocol (EMELCA Bioscience, Breda, The Netherlands). Each sample was diluted 1:10 and tested in triplicate. Deviation between triplicates was < 10% for any reported value.

Serum ghrelin levels were measured by an ELISA kit, with the lowest sensitivity threshold of 0.31 ng/ml, and the intra-assay and inter-assay CV were, respectively, 3.2 and 3.5%. For samples with serum ghrelin concentration higher than 20 ng/ml, the ELISA tests were repeated using a greater dilution factor (1:100).

Pro-opiomelanocortin (POMC) ELISA evaluation kit is based on the competitive binding enzyme immunoassay technique. The lowest sensitivity of the assay was 10 pg/ml, and the intra-assay and inter-assay CV were, respectively, 6.0 and 10.1%.

Serum Agouti-related peptide (AGRP) concentrations were measured by an ELISA kit, with sensitivity of 2.7 pg/ml; intra-assay and inter-assay CV were < 10 and < 12%, respectively.

Finally, serum leptin concentrations were measured by an ELISA kit. The lowest sensitivity was 60 pg/ml and intra-assay CV was 6.5 and 7.8%, respectively.

### Immunofluorescence assay for orexigenic and anorexigenic molecules interference

Immunofluorescence interference tests were performed by slides of frozen primate periventricular hypothalamus (Euroimmun, Lubeck, Germany), as previously described.

To understand the role orexigenic and anorexigenic molecules recognized by autoantibodies present in AN sera, ghrelin, POMC, AGRP and α-MSH previously depleted sera were used. To this end, sera were incubated for 3 h at room temperature on ELISA plates coated with recombinant human soluble molecules (10 mg/ml) of ghrelin, POMC, AGRP and α-MSH (EMELCA Bioscience, Antwerp, The Netherlands). This procedure was repeated twice before utilizing these sera in immunofluorescence assays.

After depletion, sera were serially diluted from 1:10 to 1:80 in PBS (pH 7.3), and immunofluorescence tests were performed. After incubating for 30 min at room temperature, fluorescent anti-human immunoglobulins class G (IgG) conjugate was used, as previously described.

### Statistics

Normality distribution of data was verified using D’Agostino-Pearson normality test [22]. This test computes the skewness and kurtosis to quantify how far the distribution is from Gaussian in terms of asymmetry and shape; then, it calculates how much each of these values differs from the value expected with a Gaussian distribution, and computes a single *P* value from the sum of these discrepancies.

For normally distributed data, always present, statistical analysis was performed by using the Mann–Whitney *U* test for comparison of IgG autoantibodies, ghrelin, POMC, ARGP, and leptin levels between AN patients and healthy donors.

Spearman correlation analysis was used to evaluate the relation among ghrelin, POMC, ARGP or leptin levels and anti-hypothalamic antibodies. A *p* value of less than 0.05 was considered statistically significant. All the analyses were performed by using the GraphPad Prism software 6.0 (GraphPad Software Inc., CA, USA).

## Results

### Anti-hypothalamic autoantibodies can be detected in serum from AN subjects

**Fig. 2:**
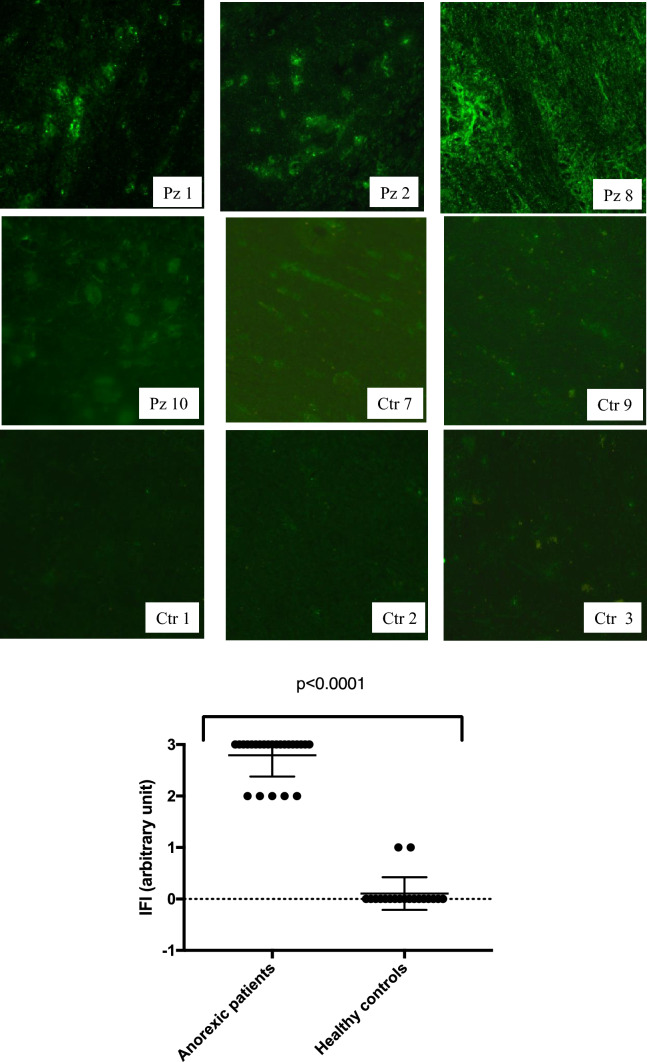
Serum from AN patients show an immune-reaction against hypothalamic cells. Immunofluorescent detection of binding of immunoglobulin G autoantibodies from 22 patients with AN to a primate hypothalamus section at the level of the arcuate nucleus (Pz 1, 2 and 8 are presented of examples of score 3 immunoreactive pattern, and Pz 10 of score 2). As control, 3 out 18 representative sera from healthy control are shown (Ctr1-3 as examples of score 0, and Ctr7 and Crt9 of score 1). The experiment was repeated four times. In the graph, the different reactivity among AN and healthy control is statistical different. The intensity of the fluorescence was determined by two different readers blind to subject characteristics and a scale from 0 to 3 was assigned (0 no reaction, 1 uncertain fluorescence, 2 nuclear fluorescence, 3 fibers and/or nuclear fluorescence). Samples scored positive if a 2 or 3 fluorescence reaction was observed

In the group of AN, all patients resulted positive for anti-hypothalamus antibodies. In detail, 15 showed cytoplasmic fluorescence, while 7 showed both cytoplasmic and fiber fluorescence (Fig. 2). Among the 18 healthy controls, 16 showed undetectable and 2 uncertain fluorescence anti-hypothalamic antibodies (Fig. 2, last row, represents a typical no reaction). Data are summarised in Tables 1, 2.

**Fig. 3:**
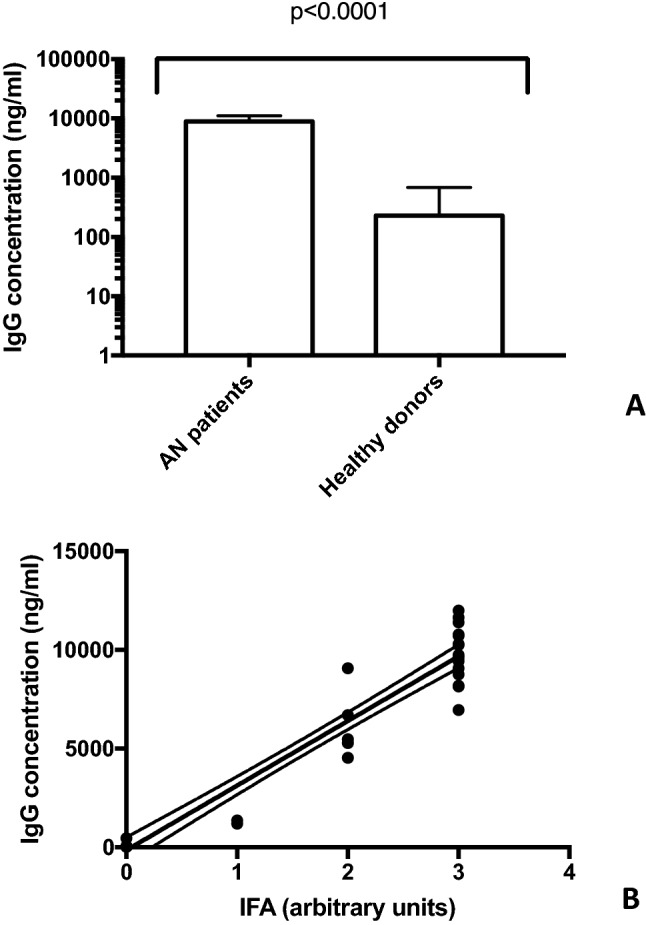
Anti-hypothalamic antibody IgG levels are present in sera from AN patients. A measurable anti-hypothalamic IgG antibody level is present exclusively in AN patients (Panel **A**). The amount of anti-hypothalamic IgG antibody correlates positively with the intensity of fluorescence on hypothalamus section (Panel **B**)

To confirm that autoantibodies from sera of AN patients were able to react with hypothalamic antigens, we performed home-made ELISA assays evaluating quantitatively serum IgG hypothalamic autoantibodies. Results are shown in Fig. 3. Concentrations of all anti-hypothalamus IgG antibodies were significantly higher in AN patients than in healthy controls (*p*<0.0001) (Fig. 3A). Healthy controls had lower levels of autoantibodies (in most cases below the sensibility level) than AN patients (mean 229.9 ng/ml ± 455.3 vs*.* 8900,0 ng/ml ± 2167.0; range 30.0–1330 ng/ml *vs*. 4534.0–11988.0 ng/ml; *p*<0.0001).

Finally, the correlation analysis among immunofluorence results and anti-hypothalamus IgG concentration was performed (Figure 3B). Of interest, a significant positive correlation between the evaluation of the presence of autoantibodies specific for hypothalamus by the two methods exists (*r*=0.934, *p*<0.0001).

Finally, no correlation among age at onset or duration of the illness and anti-hypothalamus reactivity was found (data not shown).

### Orexigenic and anorexigenic molecules evaluation

**Fig. 4:**
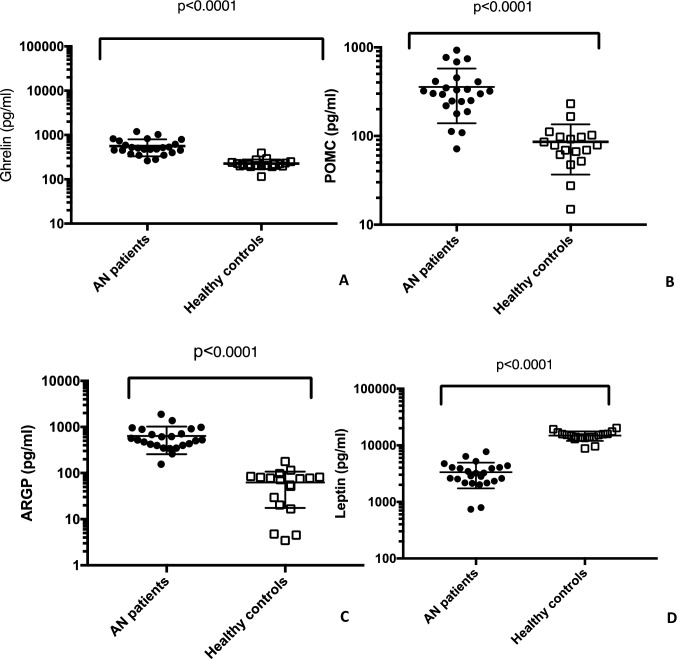
Orexigenic and anorexigenic molecules evaluation. A comparison of plasma ghrelin, POMC, AGRP and leptin levels in women with AN and healthy control women are shown

Serum levels of ghrelin in controls and AN subjects are shown in Fig. 4A. Mean serum level of ghrelin measured using ELISA was significantly higher in AN patients than controls (563.0 pg/ml ± 238.6 vs. 224.6 pg/ml ± 56.43, *p* < 0.0001; range 262.4–1200.0 pg/ml vs. 115.4–392.6 pg/ml).

Subsequently, serum levels of POMC in controls and AN patients were evaluated (Fig. 4B). Mean serum level of POMC measured using ELISA was significantly higher in AN subjects than controls (369.6 pg/ml ± 231.2 vs. 77.2 pg/ml ± 54.1, *p* < 0.0001; range 71.5–928.2 pg/ml vs. 14.9–231.2 pg/ml).

Analysis of serum levels of ARGP in controls and AN patients are shown in Fig. 4C. Mean serum level of ARGP measured using ELISA was significantly higher in AN patients than controls (664.5 pg/ml ± 402.6 vs. 59.4 pg/ml ± 49.1 *p* < 0.0001; range 155.0–1883.0 pg/ml vs. 3.5–174.2 pg/ml).

Serum levels of leptin in controls and AN patients are statistically different (Fig. 4D). Mean serum level of leptin measured using ELISA was significantly lower in AN patients than controls (3463.0 pg/ml ± 1668.0 *vs.* 14,553.0 pg/ml ± 3112 *p* < 0.0001; range 7141.1–7674.0 pg/ml vs. 8780.0–20,282.0 pg/ml).

**Fig. 5:**
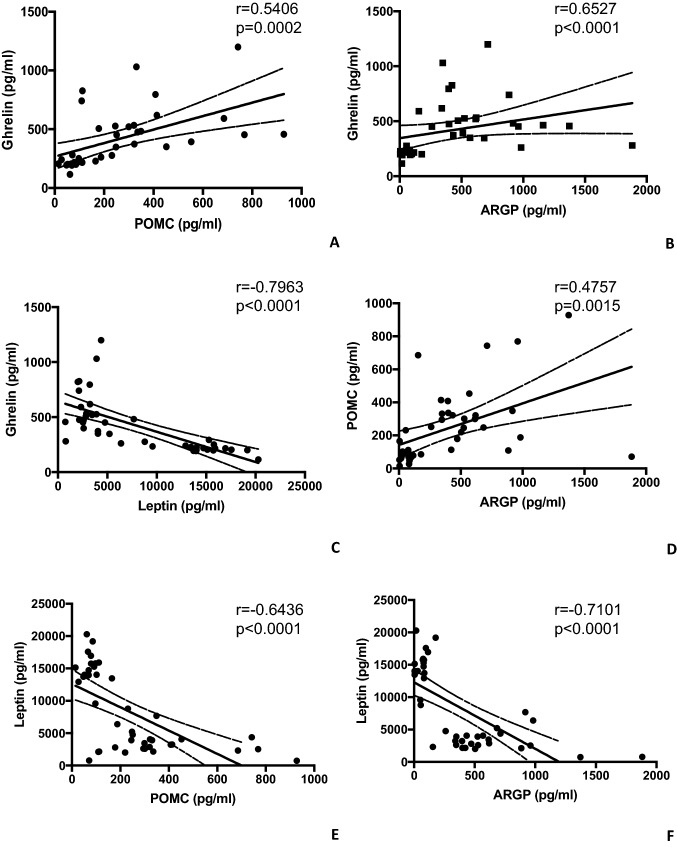
Correlation analysis among the amount of orexigenic and anorexigenic molecules was performed

Finally, the correlation analysis among different analytes was performed. A significant positive correlation was detected between serum levels of ghrelin and POMC (*r* = 0.5291, *p* < 0.0008; Fig. 5A), between serum levels of ghrelin and ARGP (*r* = 0.6313, *p* < 0.0001; Fig. 5B), and between serum levels of POMC and ARGP (*r* = 0.4497, *p* = 0.0052; Fig. 5D) in all subjects analysed. On the contrary, a significant negative correlation was detected between serum levels of ghrelin and leptin (*r* = − 0.7869, *p* < 0.0001; Fig. 5C), between serum levels of POMC and leptin (*r* = − 0.6631, *p* < 0.0001; Fig. 5E), and between serum levels of ARGP and leptin (*r* = − 0.7044, *p* < 0.0001; Fig. 5F) in all subjects analysed.

In addition, there was no correlation between the serum amount of these molecules and other blood analytes (i.e. albumin, transferrin, and insulin like growth factor-1), neither in AN subjects nor in both groups (data not shown). As a final point, there were no correlations between age at onset or duration of the illness and any blood analyte levels (data not shown).

### Orexigenic and anorexigenic molecules concentration in comparison to anti-hypothalamus antibodies

**Fig. 6:**
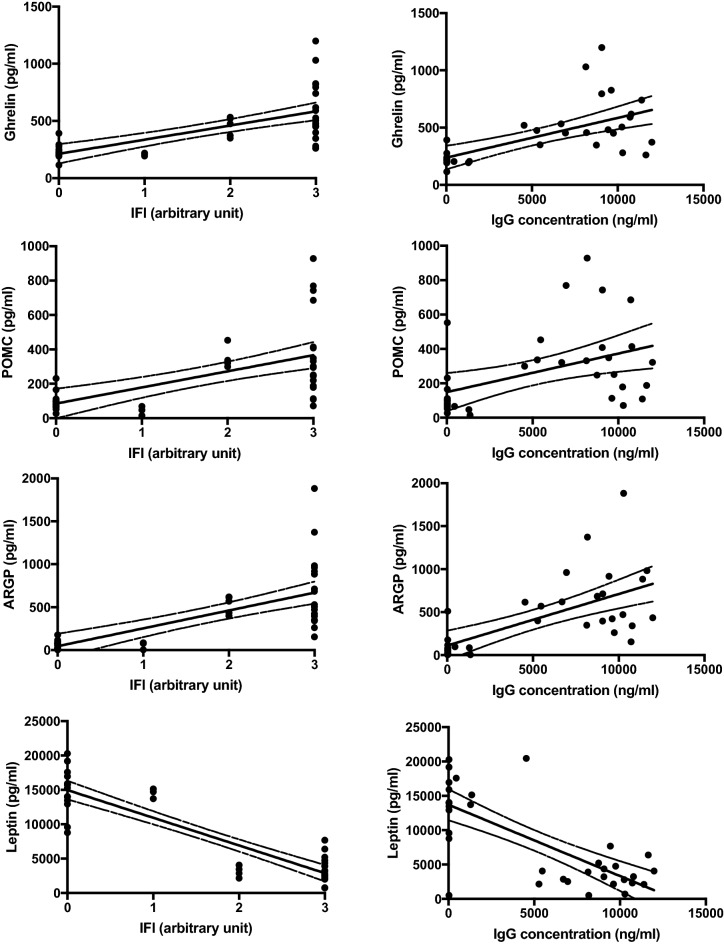
Correlation analysis among the amount of orexigenic and anorexigenic molecules and anti-hypothalamic antibodies evaluated by immunofluorescence (left panels) and ELISA (right panels)

Correlation analyses were performed between orexigenic and anorexigenic molecules concentration versus anti-hypothalamus antibodies. A significant positive correlation was detected among serum levels of ghrelin, POMC, and ARGP and the anti-hypothalamus antibodies evaluated both intensity of positive fluorescence (Fig. 6, left panels), as well as amount measured by ELISA (Fig. 6, right panels).

On the contrary, a significant negative correlation was detected between serum levels of leptin and intensity of positive fluorescence versus anti-hypothalamus antibodies (Fig. 6).

### Orexigenic and anorexigenic molecules concentration in comparison to BMI

**Fig. 7:**
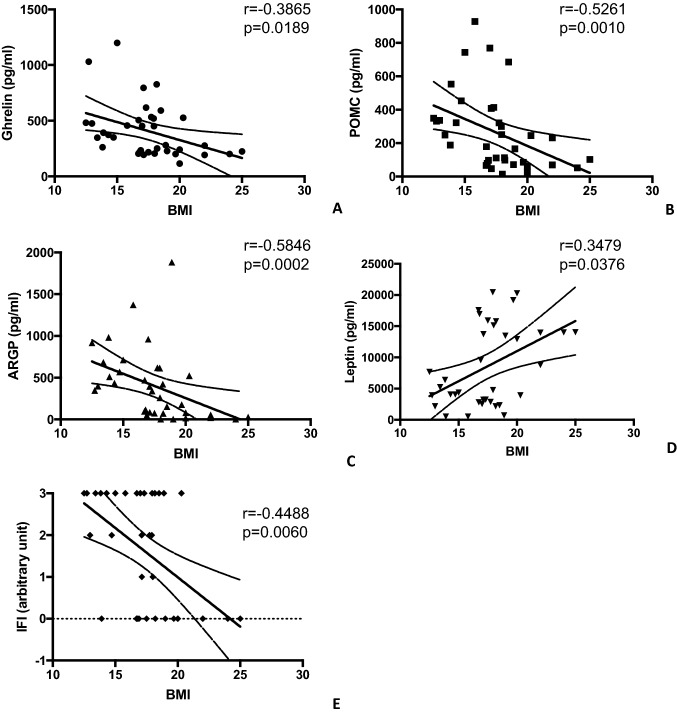
Analysis of the correlation among ghrelin, POMC, ARGP, leptin, and fluorescent intensity (IFI) with BMI

Serum amounts of orexigenic and anorexigenic molecules, as well as immunofluorescence results, were compared with the BMI of the AN patients and controls. As can be seen from Fig. 7, ghrelin, POMC and ARGP correlate negatively with the values of BMI. Meanwhile, there is a positive correlation between serum leptin values and the BMI of our study group.

In addition, a negative correlation between presence of anti-hypothalamus antibodies and the BMI exists (Fig. 7).

### Orexigenic and anorexigenic molecules are able to interfere with autoantibody assays

**Fig. 8:**
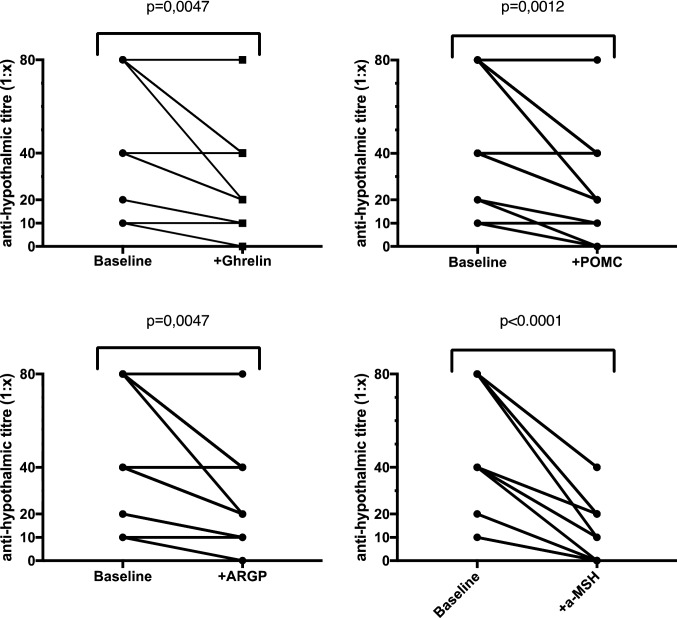
Orexigenic and anorexigenic molecules are able to interfere with autoantibodies binding. As orexigenic and anorexigenic molecules are thought to play prominent roles in integration of peripheral and central signals to modulate appetite and metabolism, experiments were set up to investigate whether they may interfere with the binding of reactive autoantibodies to hypothalamic tissues. All 22 AN sera were titred alone and in presence of recombinant human soluble molecules of ghrelin, POMC, AGRP, and α-MSH

As shown in Table 3 and in Fig. 8, sera pre-treatment with recombinant human soluble molecules of ghrelin, POMC, AGRP, and α-MSH inhibit the reaction of serum autoantibodies to hypothalamic cells in a different way. This competition effect is measurable as a reduction of the positivity titre. Of interest, immunofluorescence staining does not become totally negative (Table 3). In fact, the addition of ghrelin, POMC or ARGP significantly reduces the fluorescence intensity (Fig. 8). However, although the competition with α-MSH has a greater effectiveness, the autoantibody reaction is not completely inhibited (Table 3 and Fig. 8).

**Table 3:** Immunofluorescence titre of autoantibody reactivity on hypothalamic cells from AN serum patients, before and after depletion by recombinant human Ghrelin, recombinant human POMC, recombinant human AGRP, or recombinant human α-MSH soluble molecules

	Baseline (1:x)	+ Ghrelin (1:x)	+ POMC (1:x)	+ ARGP (1:x)	+ α-MSH (1:x)
1	80	80	80	80	40
2	40	20	20	20	10
3	40	20	40	20	20
4	80	40	20	40	10
5	40	40	20	40	20
6	40	20	20	20	0
7	40	20	20	20	10
8	40	20	20	20	20
9	80	40	40	40	20
10	40	40	40	40	10
11	20	10	0	10	0
12	80	40	40	40	20
13	80	40	40	40	40
14	80	40	40	40	40
15	10	10	10	10	0
16	10	0	0	0	0
17	20	10	10	10	0
18	80	40	20	20	20
19	80	20	40	40	40
20	80	40	40	40	40
21	80	40	40	40	20
22	80	40	40	40	20

## Discussion

Anorexia nervosa (AN) is a psychiatric disorder of complex and not fully known aetiology [1–3]. It is characterised by a lower body weight than healthy, distortion of body image and fear of gaining weight accompanied by persistent behaviours that interfere with weight gain [1–3, 17]. It is classified as an eating disorder and there is a significant risk of morbidity and mortality due to hemodynamic and electrolytic alterations or suicide [3].

The incidence of anorexia nervosa is increasing in developed countries, affecting more frequently females than males, and its beginning is usually in the adolescence years or in the early twenties [1–3]. Some studies suggest that improved living conditions increase the risk of developing this pathology [13]. Of note, improved hygiene, gross reduction of infections, and changes in dietary habits are factors potentially associated with the increase in autoimmune diseases (such as multiple sclerosis, type I diabetes mellitus, Crohn’s disease, rheumatoid arthritis, and thyrotoxicosis, in addition to asthma, eczema, and hay fever) [23]. These pathological conditions are all considered to be of autoimmune origin, or immune-mediated, and are more common in females (except type I diabetes mellitus) [24]. These characteristics suggest the possibility of an autoimmune aetiology of AN.

Fetissov et al. [25] demonstrated the presence of IgG antibodies in serums of AN patients that bind to peptidergic neurons in the rat hypothalamus and pituitary cells. These autoantibodies can specifically recognise the stimulating hormone alpha-melanocyte (α-MSH), adrenocorticotropic hormone (ACTH) and luteinizing hormone-releasing hormone (LHRH) [25]. Recent researches on intestinal microbiota–brain axis show that the bacterial protein *Escherichia coli* caseinolytic protease B, an anorexigenic bacterial protein, is responsible for the production of α-MSH cross-reactive autoantibodies [25]. Moreover, it has been shown that the binding of autoantibodies with α-MSH increases the activation of the appetite-regulating melanocortine type 4 receptor [26].

In this study, we demonstrated the presence of hypothalamus-reactive autoantibodies in the serum of AN patients through two different methods: with a qualitative immunofluorescence test on hypothalamus sections of primates and with a quantitative ELISA test. Of note, a correspondence between the two different methods was highlighted, allowing us to demonstrate a significant increase of autoantibodies specific for hypothalamus in patients with AN compared to the group of healthy donors. It is particularly interesting to note the Ig class specificity that has been measured in the serum of AN patients. In fact, while it was possible to measure IgG autoantibodies, the quantitative IgM assay gave negative results (data not shown). This may be related to the temporal function of autoantibody production in response to specific brain antigens associated with blood-brain barrier impairment [21, 27]. We can assume that the clinical definition of AN is late compared to the onset of the disease. In any case, the patients enrolled in this study have been diagnosed AN for at least 6 months before.

Of interest, sera reactivity at the level of the nerve fibers need to be further investigated. In fact, it appears that anti-neurofilament antibody levels correlate with the progression of several neurological diseases [28]. It has been speculated that neurofilament light chain could act as marker of axonal injury in different neurological disorders, including multiple sclerosis, neurodegenerative dementia, stroke, traumatic brain injury, amyotrophic lateral sclerosis and Parkinson disease [29].

Several hypothalamic antigens triggering immune responses are previously demonstrated, and α-MSH seemed to be the most important [25]. However, the competitive immunofluorescence experiments showed that some of the reactivity to hypothalamic cells may be linked to antigens not yet known. Thus, future efforts should be aimed to identify them.

To date, it is known that regulatory peptides, reacting on specific neuronal receptors in the hypothalamus, can regulate appetite [30]. Autoantibodies may have a direct effect on appetite or may affect appetite-related emotions [16].

It is not easy to determine the action of these antibodies in vivo. We can hypothesize a binding with antigens expressed by hypothalamic cells, thus preventing them from playing their physiological role. Alternatively, they could induce an aspecific stimulation of hypothalamic cells that leads to an increase secretion of anorexigenic molecules.

Ghrelin is a gastrointestinal hormone produced by enteroendocrine cells, especially of the stomach, often called “hunger hormone” [31]. Ghrelin can stimulate food intake, gastrointestinal motility, lipogenesis, blood glucose levels, lower blood pressure and inhibit the release of LH and FSH [31]. Alterations in the secretion of ghrelin may play an important role in the development of anorexia nervosa [32]. However, the mechanism by which changes in ghrelin levels correlate with weight recovery, and therefore with body mass index, are not fully clarified yet [33].

This study has demonstrated that serum ghrelin, POMC and AGRP levels are significantly increased, whereas serum leptin levels are significantly decreased in a sample of patients with AN. In addition, the levels of anti-hypothalamus antibodies measured both by reactivity to primate hypothalamic tissues and by the quantitative ELISA method correlate positively with ghrelin, POMC and AGRP levels, and negatively with leptin ones.

Leptin is an anorexigenic adipokine secreted from adipose tissue, playing a key role in homeostasis of body weight and in the psychophysiological processes associated with AN [34]. Leptin is known to have pleiotropic effects mainly affecting neuroendocrine and immune homeostasis, but also thermogenesis, reproduction, angiogenesis, and hematopoiesis [34, 35]. Serum leptin levels were found to be decreased in AN patients compared to controls (*p*<0.0001). However, this decrease appears to be reversible during weight recovery (as previously demonstrated) [31, 34].

Leptin principally acts on the hypothalamic arcuate nucleus, influencing the activities of two different groups of neurons with opposing feeding: POMC and AGRP neurons [30, 35–37]. Interaction by leptin or other molecules is possible, because these neurons have endings close to the fenestrated capillaries of the median eminence overcoming the blood-barrier. [30, 35–37]. POMC neurons, expressing leptin receptor, project to the dorsomedial, paraventricular nuclei and lateral hypothalamus [36, 37]. Thus, leptin may be the signal linking peripheral energy stores with POMC signalling activity in the hypothalamus. POMC can reduce appetite and food consumption both in the immediate and in longer time scales [38]. Leptin receptor is also expressed in several other hypothalamic nuclei that may have a role in appetite [39]. It must be remembered that not all mammalian hypothalamic POMC neurons express leptin receptors [39, 40], suggesting a possible existence of POMC neurons signalling activity leptin-unrelated too.

AGRP neurons produce the agouti-related peptide (AGRP) which is one of the most powerful appetite stimulators [41]. The role of elevated plasma AGRP levels in AN remain unclear [41]. It can be assumed that changes of AGRP levels in AN might be part of an extensive adaptive response to the negative energy balance involving other substances regulating food intake and energy expenditure such as ghrelin, leptin, α-MSH and POMC [30]. However, function of peripherally circulating orexigenic and anorexigenic molecules, as well as the presence of autoantibodies to hypothalamic neurons is not fully known. Thus, more extensive study is required.

## Limits and strengths of the study

We are aware of the possible limitations of immunofluorescence because of its poor specificity or even false positivity. Otherwise, we are aware that the assessment of the positivity to immunofluorescence is dependent on “visual judgement” (microscope observation). For all these reasons, ELISA test was performed to support the results. In addition, we are planning to continue the study using human tissues to better highlight autoantibody reactions.

## Conclusion

In this pilot study, we analysed the serum of 22 patients with AN and a selected control group of 18 patients matched for age and sex. For all AN patients analysed we demonstrated an immuno-reactivity towards primate hypothalamic neurons. This was not evident for any of the control group sera. Therefore, these results appear to confirm the presence of autoantibodies associated with AN. In addition, it could be envisaged that the presence of autoantibodies could interfere with neuron–neuron interaction in the cascade of appetite controls.

### What is already known on this subject?

It has recently been speculated that AN may be a disease linked to a dysregulation of the immune system. Autoantibodies specific for neuropeptides, neurotransmitters and peptide hormones regulating appetite could be involved in AN.

### What the study adds?

The presence of autoantibodies to hypothalamic cells in the serum of AN patients was verified by two different methods (one qualitative and one quantitative).

The levels of anti-hypothalamus autoantibodies measured both by reactivity to primate hypothalamic tissues and by quantitative ELISA method correlate positively with ghrelin, POMC and AGRP levels, but negatively with leptin ones.

Finally, competition experiments with recombinant molecules have shown that in addition to ghrelin, POMC, ARGP and α-MSH, other unknown target molecules for autoantibodies present in AN serum may exist.

## Data Availability

All data are available.
